# Coumarins and P450s, Studies Reported to-Date

**DOI:** 10.3390/molecules24081620

**Published:** 2019-04-24

**Authors:** Maryam Foroozesh, Jayalakshmi Sridhar, Navneet Goyal, Jiawang Liu

**Affiliations:** 1 Department of Chemistry, Xavier University of Louisiana, 1 Drexel Dr., New Orleans, LA 70125, USA; jsridhar@xula.edu (J.S.); Ngoyal@xula.edu (N.G.); 2 Department of Pharmaceutical Sciences, College of Pharmacy, University of Tennessee Health Science Center, 881 Madison Ave., Memphis, TN 38163, USA; jliu90@uthsc.edu

**Keywords:** cytochrome P450, coumarins, docking, quantitative structure-activity relationship (QSAR), hologram quantitative structure-activity relationship (HQSAR), comparative molecular field analysis (CoMFA), comparative molecular similarity index analysis (CoMSIA), molecular modeling, active site, competitive inhibition, time-dependent inhibition

## Abstract

Cytochrome P450 enzymes (CYPs) are important phase I enzymes involved in the metabolism of endogenous and xenobiotic compounds mainly through mono-oxygenation reactions into more polar and easier to excrete species. In addition to their role in detoxification, they play important roles in the biosynthesis of endogenous compounds and the bioactivation of xenobiotics. Coumarins, phytochemicals abundant in food and commonly used in fragrances and cosmetics, have been shown to interact with P450 enzymes as substrates and/or inhibitors. In this review, these interactions and their significance in pharmacology and toxicology are discussed in detail.

## 1. Introduction

### 1.1. Cytochrome P450s Enzymes

Cytochrome P450 enzymes (CYPs), an important superfamily of Phase I enzymes, are involved in the metabolism of endogenous and exogenous (xenobiotic) compounds [1,2,3,4]. These versatile enzymes are found in all living organisms and catalyze the mono-oxygenation of a wide variety of substrates [1]. The primary purpose of P450s is detoxification by oxidizing non-polar molecules into more polar and excretable ones [5]. Mammalian P450s play important roles in cholesterol and hormone synthesis, metabolism of endogenous compounds such as vitamin D and bile acids, drug activation and deactivation, and xenobiotic detoxification [1,2,3,4]. About 75% of known enzymatic reactions on drugs are catalyzed by P450 enzymes [6]. In addition, these enzymes play an important role in carcinogenesis, as certain pro-carcinogenic compounds are metabolized by P450 enzymes into their ultimate carcinogenic forms, which are capable of binding DNA and initiating cancer [1,7,8]. P450s are estimated to catalyze about 66% of bioactivation reactions, many of which lead to cancer formation [9,10,11].

The Human Genome Project has shown that there are 57 human P450 genes and 58 pseudogenes [1,12]. As more substrates including environmental chemicals and drugs have been discovered for the various P450 enzymes, their versatility and importance have become more apparent, warranting additional studies on their mechanisms of action and roles in different diseases including cancer. The development of selective inhibitors for P450 enzymes involved in carcinogenesis has been the focus of many research groups, including ours and our collaborators [13,14,15,16,17,18,19,20,21,22,23].

#### 1.1.1. Basic Enzyme Structure 

P450 Enzymes are a superfamily of hemoproteins [1]. Even though the amino acid sequence identity across the entire P450 superfamily is less than 20%, the enzymes have identical overall fold structures. The three-dimensional structure of P450s consists of 12 α-helices (A-L), forming the bulk of the protein, in addition to four β-sheets. The heme is located within the L-helix and the highly conserved I-helix that is perpendicular to the F/G segment made from the F-helix, F/G-loop and the G-helix [23]. The heme-Fe atom is bound to the sulfur atom of the cysteine amino acid in the adjacent loop, which contains the highly conserved sequence FxxGx(HRK)xCxG [23]. Substrate access and specificity for the various P450 active sites are determined by the B-C and F-G helices [23]. Crystal structures of many P450 enzymes have already been published [24,25,26,27,28,29,30,31]. 

#### 1.1.2. Catalytic Activity Cycle

**Figure 1:**
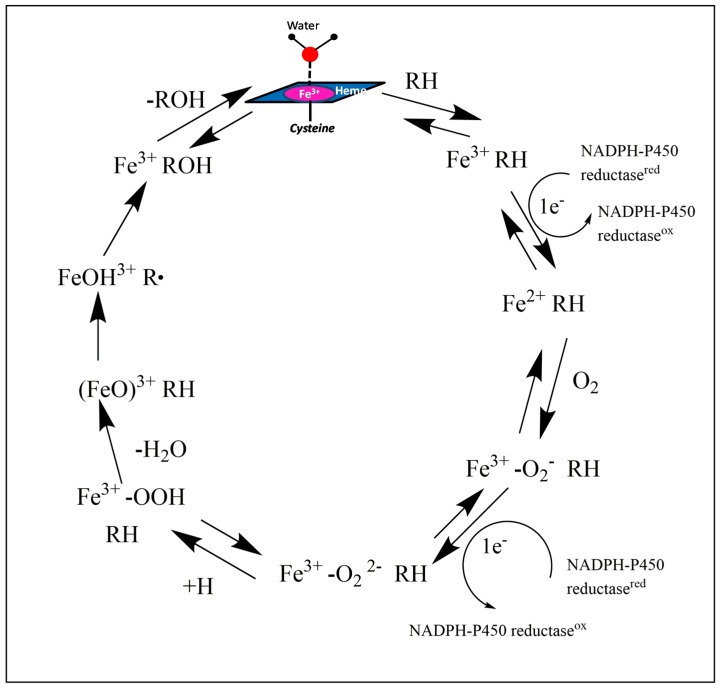
The catalytic oxidation cycle of cytochrome P450 enzymes—The heme-Fe atom is covalently bound to the sulfur atom of a cysteine amino acid residue in the enzyme active site [23].

The catalytic activity starts once a substrate is bound to the enzyme, and the low-spin ferric heme-iron (coordinated to a water molecule as its sixth ligand in its resting state) changes to a high-spin ferric state in the enzyme-substrate complex. The heme-iron then binds an oxygen molecule to form a stable oxy-iron-enzyme complex. This oxy-iron-enzyme complex is then reduced to form an unstable peroxo-ferric-enzyme complex, followed by the protonation of the distal oxygen to form an unstable hydroperoxo-iron-enzyme complex. A second protonation of the same oxygen is then followed by heterolytic cleavage of the bond between the two oxygen atoms leading to the formation of a reactive high-valent iron-oxo-enzyme complex and water. This complex then abstracts a hydrogen atom from the substrate with subsequent radical recombination to form the product (oxidized form of the substrate). After the release of the product, water binds to the heme-iron, regenerating the resting P450 enzyme (Figure 1) [1,23].

### 1.2. Coumarins

**Figure 2:**
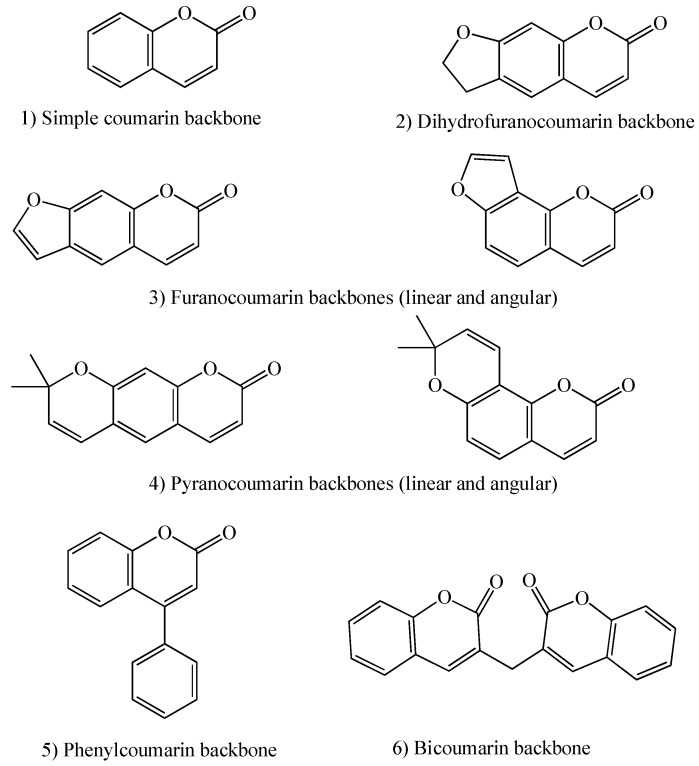
Types of coumarin backbone structures.

Coumarins are natural products found in plants, fungi, and bacteria [32]. They are commonly found in food and also widely used in cosmetics and fragrances [33,34]. Coumarins show a variety of biological activities, including anti-oxidant, anti-cancer, anti-coagulant, anti-inflammatory, anti-fungal, anti-microbial, anti-viral, anti-neurodegenerative, and anti-diabetic activities [32,33,34]. Coumarin (2H-1-benzopyran-2-one) backbone structure is made of fused benzene and α-pyrone rings [35,36]. This conjugated structure also leads to coumarins’ applications as fluorescent sensors for biological activities [32]. There are six types of coumarins based on their extended backbone structures: 1) Simple coumarins; 2) Dihydrofuranocoumarins; 3) Furanocoumarins (linear and angular); 4) Pyranocoumarins (linear and angular); 5) Phenylcoumarins; and 6) Bicoumarins (Figure 2) [36]. 

#### 1.2.1. Coumarins and P450s 

Due to the importance of understanding the interactions between coumarins and cytochrome P450 enzymes and their biological effects, especially on human health, this review is focused on studies of such interactions and lessons learned. 

The P450 enzymes have a wide range of natural product substrates and inhibitors including coumarins [37,38,39,40,41]. For example, citrus fruits are rich in coumarins such as bergamottin, a linear furanocoumarin found in grapefruits, that potently inhibits several P450 enzymes including P450s 1A1, 1A2, 2A6, 2B6, 2D6, 3A4, and 3A5 [42,43]. These interactions are especially important in pharmacology as many of these P450s are required for bioactivation and/or metabolism of drugs in vivo, and their inhibition can lead to lower bioavailability of the drugs’ active metabolites and/or higher concentrations of the drugs in the blood or tissues that are prone to drug toxicity. Furanocoumarins are secondary metabolites in citrus plants and protect the plants against other organisms including insects [41]. Interestingly their biosynthesis in plants is also heavily P450 dependent [44]. A new X-ray crystal structure of P450 1A1 with bergamottin in its active site has been published recently, showing that the P450 1A1 active site is malleable and opens up as needed to accommodate large substrates [28]. This specific P450 enzyme is heavily involved in human carcinogenesis, thus its inhibition has been studied for cancer chemoprevention [28]. Bergamottin has been shown to have chemo-preventive effects in mice, inhibiting benzo[a]pyrene metabolism by P450 1A1 to its carcinogenic form, anti-benzo[a]pyrene-7,8-diol-9,10-epoxide, and the resulting production of DNA adducts and cancer formation [45]. Another naturally occurring linear furanocoumarin, methoxsalen, is a very potent mechanism-based, non-selective inhibitor of human P450s, and is used as a drug for the treatment of a number of skin diseases such as psoriasis [46,47]. Two other linear furanocoumarins, imperatorin and isopimpinellin, have been shown to have anti-cancer activities in a murine breast cancer model through the inhibition of P450 1A1/1B1 metabolism of 7,12-dimethylbenz[a]anthracene (DMBA) and blocking DMBA-DNA adduct formation [48].

**Figure 3:**
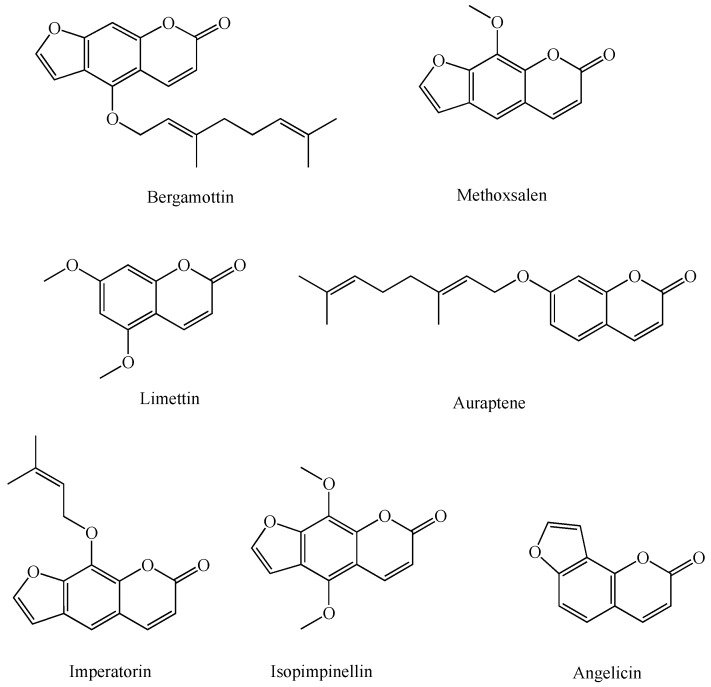
Sample coumarin P450 substrates and inhibitors.

Figure 3 contains the structures of a number of natural coumarin P450 substrates and inhibitors [45]. Coumarins exhibit significant species differences in metabolism and toxicity [37,40].

#### 1.2.2. Coumarin Metabolism by P450s

**Figure 4:**
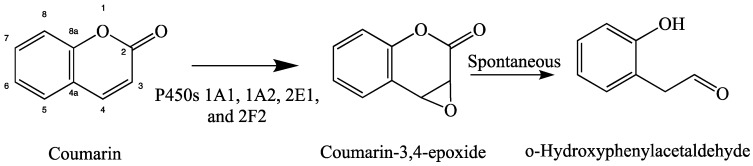
Coumarin metabolism leading to toxicity in rodents.

As mentioned previously, P450 metabolism of coumarins is species dependent. Coumarin has been found to be a rat liver and mouse lung toxicant due to the P450-dependant production of coumarin-3,4-epoxide by P450s 1A1, 1A2, and 2E1 in liver and P450 2F2 in lung (Figure 4) [37,49]. 3-Hydroxycoumarin has been found as a minor metabolite of coumarin metabolism by recombinant rat P450s 1A1 and 1A2 [37].

In humans, only about a dozen of the 57 *CYP* genes, those belonging to *CYP* families 1, 2, and 3, are involved in the metabolism of xenobiotics including about 80% of current clinical drugs [50]. Expression of these genes to corresponding enzymes (P450s 1A1, 1A2, 1B1, 2A6, 2A13, 2B6, 2C8, 2C9, 2C19, 2D6, 2E1, 2J2, 3A4, and 3A5) is influenced by combinations of different factors including genetic polymorphisms, sex, age, ethnicity, general health conditions, and induction by xenobiotics [50].

The human P450 1 family has three enzymes from two subfamilies, P450s 1A1 and 1A2 from subfamily 1A and P450 1B1 from subfamily 1B. P450s 1A1 and 1B1 are primarily extrahepatic enzymes while P450 1A2 is mainly found in the liver. With 72% amino acid sequence similarity, the enzymatic activities of P450s 1A1 and 1A2 greatly overlap and mainly include the hydroxylation and oxidation of aromatic compounds including polycyclic aromatic hydrocarbons (PAHs). Coumarin is metabolized by these human enzymes into comarin-3,4-epoxide at a much lower rate than observed in rodents, and thus does not cause the same high toxicity [49]. P450 1B1 has relatively low amino acid sequence similarity with both P450 1A1 and P450 1A2, 38% and 37% respectively, however, it in general has a similar active site cavity shape and size (441 Å3 for 1B1, 469 Å3 for 1A2, and 524 Å3 for 1A1) leading to significant substrate specificity overlap with these enzymes (such as PAHs, heterocycle aromatic amines, and estradiol) [24]. P450s 1A1, 1A2, and 1B1 do not show much coumarin 7-hydroxylase activity. P450 1B1 also does not show coumarin 3,4-epoxidase activity. 3-Hydoxycoumarin has been shown to form as a minor metabolite during the incubation of coumarin with recombinant human P450 1A1 or P450 1A2 [37]. All three enzymes show 7-alkoxycoumarin dealkylation activities [48]. The order of rates of 7-ethoxy-4-trifluoromethylcoumarin deethylation by these three enzymes has been shown to be P450 1A1 > P450 1B1 > P450 1A2 [51].

**Figure 5:**
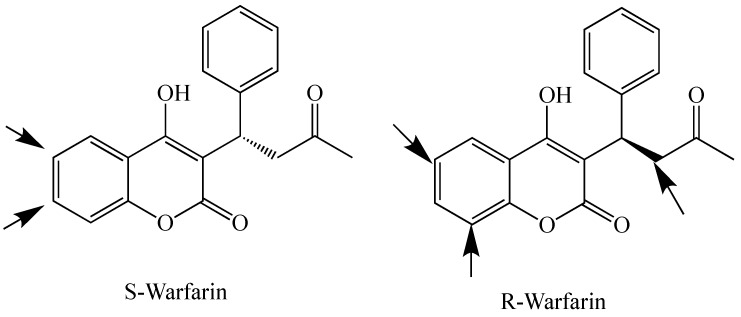
Sites of warfarin hydroxylation by P450s.

There are 16 *CYP*2 genes in humans, and most show high levels of genetic polymorphisms [50]. From the *CYP*2A subfamily, only *CYPs* 2A6 and 2A13 are functional and both show significant genetic polymorphisms. P450 2A6 is mainly hepatic, while P450 2A13 is mainly expressed in the respiratory tract. Most substrates for these enzymes, which have 94% amino acid sequence similarity, are non-planar, low molecular weight compounds which contain two hydrogen bond acceptors [50]. The two only differ in 32 amino acids, ten of which are located in their relatively small active sites (<300 Å3) [8,24]. P450 2A6 is responsible for the metabolism of about 3% of clinically used drugs (such as disulfiram, halothane, and tegafur) in addition to the metabolism and bioactivation of tobacco nitrosamines (nicotine and NNK (4-methylnitrosamino-1-3-pyridyl-1-butanone)) [50]. Polymorphisms in P450 2A6 are responsible for individual differences in the rate of nicotine metabolism, smoking behavior, and cancer risk associated with tobacco use [50]. P450 2A13 is similar in substrate specificity in general. However, it is much more efficient in the bioactivation of NNK [50]. Both enzymes are known to catalyze coumarin 7-hydroxylation and 7-alkoxycoumarin dealkylation [29,50]. The deethylation of 7-ethoxycoumarin and the demethylation of 7-methoxycomarin have been shown to produce both 7-hydroxycoumarin and 3-hydroxycoumarin as products, though the 3-hydroxylation was observed at a greater extent during the deethylation reaction [29,52]. Since position 7 has been shown to be the closest to the heme-iron, the production of the 3-hydroxy product implies rotation of the substrate during the reaction to produce this product [53,54]. P450 2A6 is the major coumarin 7-hydroxylase in the human liver, and the X-ray crystal structure of the enzyme-substrate complex has been published showing a tight fit of the coumarin molecule in the small P450 2A6 active site (260 Å3) [29,30]. Neither 2A6, nor 2A13, produce 3-hydroxycoumarin during oxidation of coumarin [29]. From the *CYP*2B subfamily, only *CYP*2B6 is functional in humans [55]. P450 2B6 has been shown to efficiently O-deethylate 7-ethoxy-4-trifluoromethylcomarin [50]. Mammalian P450s 2B35 and 2B37 from the desert woodrat (*Neotoma lepida*) have been studied for O-dealkylation activities with series of 7-alkoxycoumarins, 7-alkoxy-4-trifluoromethylcoumarins, and 7-alkoxy-4-methylcoumarins with a C1-C7 side chain, and have been shown to display differences in their substrate selectivity based on the length of the O-alkyl group [31]. P450 2B35 shows higher activity in O-dealkylation of long-chain coumarin derivatives (7 and 6 carbon chains), while P450 2B37 shows preference for short-chain coumarin derivatives (such as 7-ethoxy-4-trifluoromethylcoumarin) [31]. The crystal structures of these two enzymes in complex with 4-(4-chlorophenyl)imidazole (4-CPI) have confirmed that P450 2B35 fits two 4-CPI molecules in its active site, while P450 2B37 only fits one in the active site, though it has an open conformation with two more 4-CPI molecules in its substrate access channel [31]. Rabbit P450 2B4 has a 78% amino acid sequence similarity with human P450 2B6. Crystal structures of P450s 2B4 and 2B6 have been shown to only contain one 4-CPI molecule in their active sites [56,57]. P450s 2B4 and 2B6 also show coumarin substrate selectivity based on the side chain length at position 7, as well as substitution at position 4 [55]. These observations confirm that P450 2B family enzymes show significant differences within their flexible active sites and substrate access channels across various species, and bind a wide range of ligand sizes and shapes [31]. The *CYP*2C subfamily members in humans are *CYPs* 2C8, 2C9, 2C18, and 2C19; though 2C18 mRNA is not efficiently translated to protein, and thus this enzyme is not expressed in high concentrations [50]. Polymorphisms in these genes significantly affect drug metabolism. P450 2C9 is the main enzyme from this subfamily involved in the metabolism of coumarins, and polymorphisms have been shown to lead to coumarin sensitivity and toxicity, especially for patients on coumarin anti-coagulants (such as warfarin, acenocoumarol, and phenprocoumon) [58]. Warfarin is used as a racemic mixture of R and S enantiomers, however, the S enantiomer is 2–5 times more potent. Both enantiomers are hydroxylated by P450 enzymes during metabolism leading to their excretion in urine (Figure 5). S-warfarin is mainly metabolized by P450 2C9 to the 6-, and 7-hydroxy metabolites which are easily excreted and limit the drug’s toxicity. R-warfarin is metabolized by P450s 1A1, 1A2, and 3A4, so one enzyme containing a polymorphism does not have as much effect [59]. *CYP*2D6 is the only active gene from this subfamily in humans [50]. P450 2D6 is non-inducible, however, its hepatic concentration varies significantly between individuals due to polymorphisms [60]. This enzyme is involved in the metabolism of 15–25% of clinically used drugs, thus its genetic polymorphisms play important roles in the upregulation or downregulation of their pharmacological and toxicological effects [50]. P450 2D6 has been shown to perform 7-dealkylation reactions on 7-alkoxycoumarins such as 7-methoxy-4-aminomethylcoumarin [61]. Human P450 2E1 is an inducible enzyme and mainly metabolizes small polar molecules such as ethanol but also has some larger aromatic substrates including coumarins. P450 2E1 metabolism of ethanol leads to the formation of reactive oxygen species leading to liver cell damage in heavy drinkers [50]. Metabolism of coumarins by this enzyme leads to epoxidation at the 3,4 positions, dealkylation at the 7 position, and/or hydroxylation at different positions as previously discussed [37,49,62]. In addition, human P450 2F1 also efficiently catalyzes 7-ethoxycoumarin O-deethylation [63].

From the P450 family 3, the main human genes are *CYP*3A4, *CYP*3A5, *CYP*3A7, and *CYP*3A43. However, only P450s 3A4 and 3A5 are important in the metabolism of xenobiotics and clinical drugs, both of which show high levels of polymorphism. Tacrolimus, an immunosuppressant drug used in transplant patients, is metabolized by P450 3A5; thus, P450 3A5 genetic polymorphism testing is important to determine the correct dosage based on the patients’ rate of drug metabolism. P450s 3A4 and 3A5 have large active sites (~1400 Å3), over 85% amino acid sequence similarity, and are known to metabolize large lipophilic molecules [50]. These enzymes debenzylate 7-benzyloxy-4-trifluoromethylcoumarin, and their activities can be inhibited by some natural coumarins such as bergamottin leading to drug interactions [45,46,47,48,50,64].

**Figure 6:**
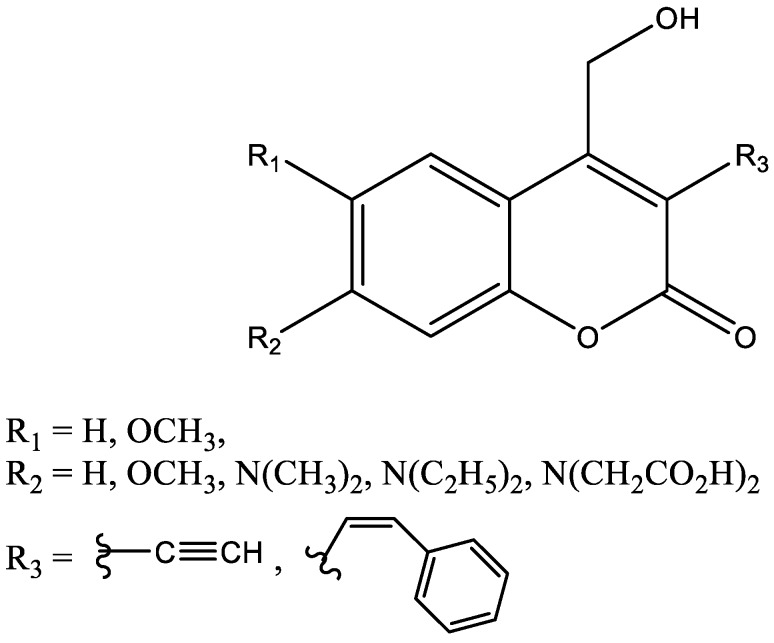
Structures of coumarin caging molecules [68,70].

Recent explorations of controlled drug delivery have pointed to engineering of photo-responsive nanoparticles as site targeted drug delivery systems [65,66,67]. These engineered nanoparticles should have the ability to hold on to the biologically active molecule (drug) until they reach the targeted site, where upon the irradiation of the photo-responsive group (“photo-cage” or “caging group”) the molecule in its active state could be released [68,69]. Due to their water solubility, versatility of their incorporation into varied types of nanoparticles, and their absorption in the visible part of the spectrum, coumarins have elicited extreme interest in this area. The photo-responsive properties of coumarins have made them the photo-cage molecule of choice for the recent studies on drug delivery. One of the main limitations of such strategy is the toxicity/drug interactions of the coumarins and the products of metabolism of the released coumarins by cytochrome P450 enzymes. Figure 6 shows the coumarin structures that have shown promise in photo-caging [68,70].

#### 1.2.3. P450 Inhibition Mechanism by Coumarins

P450 inhibition follows two distinct mechanisms, direct competitive inhibition and time-dependent inhibition. Direct competitive inhibitors are substrate-like molecules capable of entering and temporarily binding to the enzyme active site while being metabolized; once the metabolites are released, the enzyme returns back to its free state. For these compounds to be effective inhibitors, they have to show higher affinity toward the enzyme than its natural substrates, thus keeping the enzyme busy and reducing its substrate turn-around rate. Time-dependent inhibitors, also often resemble natural substrates in structure, giving them access to the enzyme active site; in addition, enzyme incubation in their presence leads to increased inhibition. Mechanism-based (suicide) inhibitors are time-dependent inhibitors that are metabolized by the enzyme to reactive intermediates which covalently bond the active site residues and permanently inhibit the enzyme’s activity [71,72,73]. This process is both time- and cofactor-dependent. Coumarins can act as substrates, direct competitive inhibitors, or time-dependent inhibitors.

Our group and collaborators have focused on the development of selective mechanism-based inhibitors for certain P450 enzymes involved in carcinogenesis including a number of coumarin derivatives [13,14,15,16,17,18,19,20,21,22,23,38,74]. P450s 1A1, 1A2, 1B1, 2A6, 2E1, and 3A4 are responsible for about 77% of all bioactivation reactions catalyzed by P450s, thus their inhibition has been studied as a potential means of cancer prevention [1,13,16,18,20,21,24,25,74,75]. However, P450 inhibitors have the potential for inducing the over-expression of P450 enzymes by activating the aryl hydrocarbon receptor (AhR) limiting their use as clinical cancer preventive agents. An ideal P450 inhibitor for use in cancer prevention should not cause AhR activation. Mechanism-based inhibitors could also be used as probes of the enzyme active site structure and reaction mechanisms [1].

**Figure 7:**
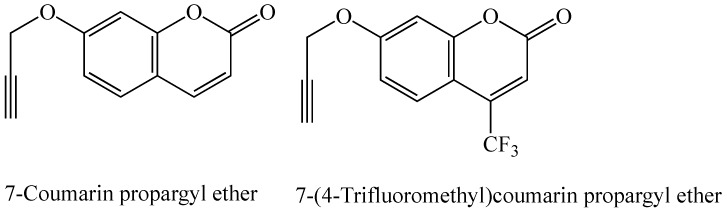
Structures of two coumarin cytochrome P450 3A4 mechanism-based inhibitors [38].

P450 3A4 is involved in the bioactivation of environmental procarcinogens such as aflatoxin B1, aflatoxin G1, and dihydrodiol derivatives of PAHs [76]. 7-Coumarin propargyl ether and 7-(4-trifluoromethyl)coumarin propargyl ether (structures shown in Figure 7) were designed and synthesized by our group, combining the structural features of a known substrate for P450s 3A4 and 3A5, 7-benzyloxy-4-trifluoromethylcoumarin (BFC), with the propargyl functional group previously shown to have a potential inhibitory effect on P450s [38]. When correctly oriented, the terminal triple bond is oxidized by the enzyme into a reactive ketene intermediate through a 1,2-hydrogen shift, leading to the formation of a covalent bond with a nucleophilic amino acid residue in the active site and loss of enzymatic activity [1,20]. Both compounds in this study inhibited the BFC O-debenzylation activity of P450 3A4 in a mechanism-based manner (time-, concentration-, and NADPH-dependent) [34]. Neither compound showed mechanism-based inhibition of BFC O-debenzylation by P450 3A5, showing selectivity for P450 3A4 [38]. This observed variation in the catalytic activity of the two enzymes with these coumarin derivatives has been attributed to their difference in 78 amino acids (out of the total 503 in their sequences), of which 17 are located in their substrate recognition sites (SRSs) leading to different ligand-enzyme interactions [38].

**Figure 8:**
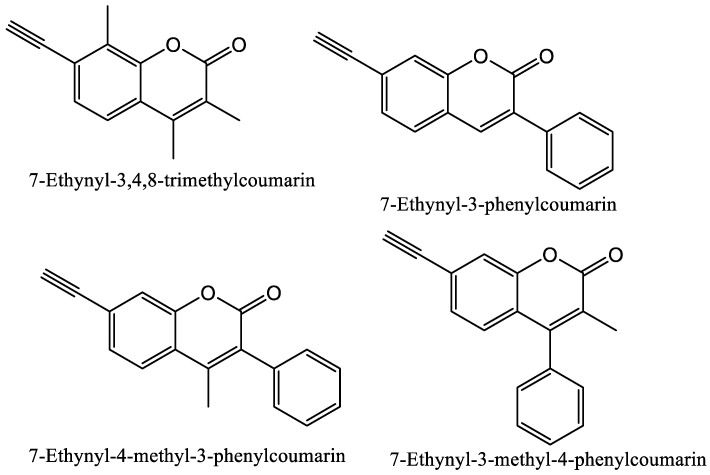
Structure of coumarin mechanism-based inhibitors with selectivity toward P450s 1A1 and 1A2 compared to P450s 2A6 and 2B1 [22].

As previously discussed, P450s 1A1 and 1A2 are involved in the bioactivation of many environmental chemicals such as PAHs [1,74,75]. P450 1A1 prefers larger planar molecules such as benzo[a]pyrene and DMBA, while P450 1A2 prefers smaller planar triangle-shaped molecules including aryl and heterocyclic amines [74,75]. A series of 7-ethynylcoumarin derivatives were designed, synthesized, and evaluated by us for potential inhibitory effects and selectivity toward P450s 1A1, 1A2, 2A6, and 2B1 [22]. These compounds were also designed by combining the coumarin backbone, a known P450 substrate, with the ethynyl functional group previously shown to cause mechanism-based inhibition [1,38]. All of the compounds in this study, except 7-ethynyl-3-methyl-4-phenylcoumarin (7E3M4PC) showed mechanism-based inhibition of P450s 1A1 and 1A2, and no inhibition of P450s 2A6 and 2B1 [22]. The most potent and selective compound in the study was 7-ethynyl-3,4,8-trimethylcoumarin (structure shown in Figure 8) [22]. Methoxsalen has been shown to inhibit P450 2A6 but lacks selectivity as it also inhibits other P450 enzymes such as P450s 3A4 and 2D6. 5-Methoxycoumarin and 6-methoxycoumarin have been shown to inhibit P450 2A6 selectively (IC_50_ values of 0.13 and 0.64 mM, respectively) [77].

**Figure 9:**
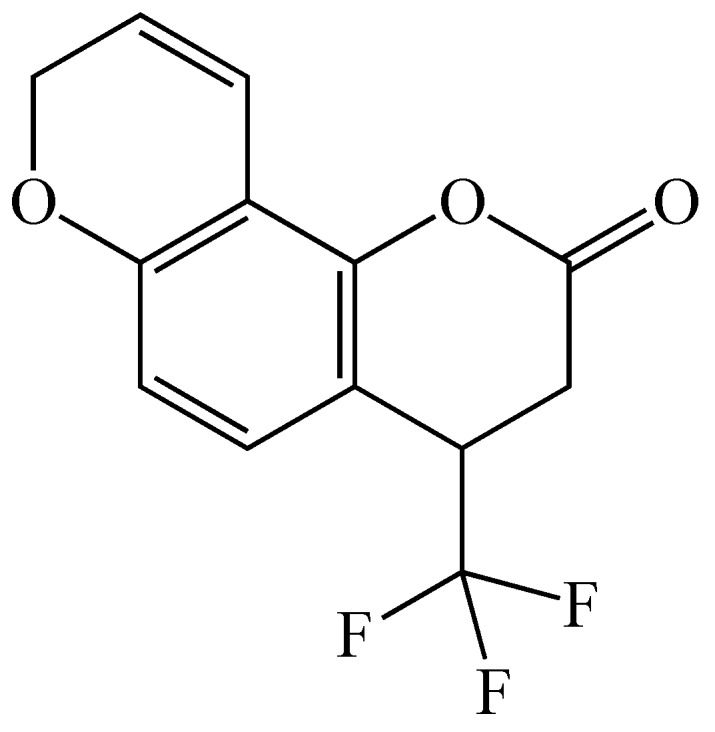
Structure of 4-trifluoromethyl-7,8-pyranocoumarin.

In a more recent study, our group designed and studied a number of furano-, pyrano-, pyridino-and dioxolo-coumarin derivatives as potential inhibitors for P450 1A2 [63]. One of the compounds in this study, 4-trifluoromethyl-7,8-pyranocoumarin, was found to be a competitive inhibitor, showing high selectivity for the inhibition of this enzyme, with selectivity indices of 155 and 52 for P450 1A2 over P450s 1A1 and 1B1, respectively (structure shown in Figure 9) [63]. This selectivity is of importance since most known P450 1A2 inhibitors also inhibit P450s 1A1 and 1B1 (80% and 40% amino acid sequence similarity, respectively). In yeast AhR activation assays, at concentrations lower than 1 μM, this pyranocoumarin did not activate the receptor suggesting that it would not up-regulate AhR-caused P450 enzyme expression [63]. These results show that 4-trifluoromethyl-7,8-pyranocoumarin is selective for the inhibition of P450 1A2 and has the potential for use in cancer-prevention [63].

#### 1.2.4. Computational Molecular Modeling Studies

**Figure 10:**
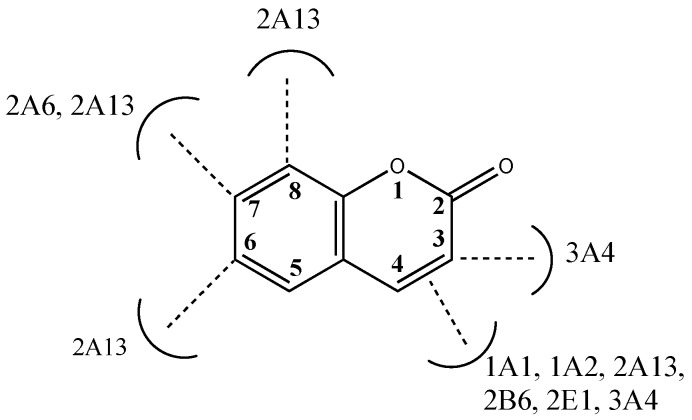
Oxidation of coumarin at various positions by different cytochrome P450 enzymes [78,79,80,81,82,83,84,85,86,87].

Coumarin metabolism studies have shown that sites of oxidation are species dependent and human P450 enzyme oxidation of coumarins mainly produces 7-hydroxycoumarin, with four other products formed only in minor amounts due to 3,4-epoxidation, and hydroxylation at the 3-, 6-, or 8- positions (Figure 10) [78,79,80,81,82,83,84,85,86,87]. Kinetic studies have been used to show that P450s 1A1, 1A2, 2B6 and 2E1 catalyze metabolism of coumarin to its 3,4-epoxide which then leads to the formation of the *ortho*-hydroxyphenylacetaldehyde as the metabolite [37,82]. P450 2A6 oxidizes coumarin at the 7-position leading to the formation of the 7-hydroxycoumarin metabolite [37,82]. P450 3A4 metabolism leads to the formation of two metabolites, namely, 3-hydroxycoumarin and *ortho*-hydroxyphenylacetaldehyde, through the coumarin 3,4-epoxidation pathway [82]. Computational molecular modeling tools have been used by several research groups to understand the interactions of coumarin molecules with the P450 enzymes. Studies have included docking studies of coumarins in the active sites of the P450 enzymes. Quantitative structure-activity relationship (QSAR) studies have provided important information on structural features of coumarins as P450 substrates [19,22,39,61,83,84]. The binding modes of coumarins vary depending on the nature and position of substituents and the P450 enzyme binding cavity topography [13,23]. Docking studies have been performed by Lewis et al. in order to understand the role of P450 binding cavity residues in the oxidative pathways of coumarin [39]. One of the key interactions shown by coumarins is the π-π stacking interactions with the phenylalanine residues in the binding pocket [39,84]. Additionally, interactions with a hydrogen-bonding residue determine the binding orientation of coumarin in the binding pocket and its site of oxidation [39,84]. The differences in spatial arrangements of the hydrogen-bonding residues and the phenylalanine residues have been shown to result in differences in the binding mode and product formation. For example, in P450 2A6, the Asn290 forms a hydrogen bond with the coumarin carbonyl group, resulting in the orientation of the 7-position towards the heme-Fe, resulting in the formation of 7-hydroxycoumarin [39]. In P450 3A4, the hydrogen bonding of the coumarin carbonyl with a Ser119 in the binding pocket leads to the 3-position facing the heme-Fe, and the formation of the 3-hydroxycoumarin [39].

Coumarin is the marker substrate for the cytochrome P450 2A family, consisting of P450s 2A6 and 2A13. As previously mentioned, these enzymes have 94% amino acid sequence identity. The catalytic efficiency of coumarin 7-hydroxylation was found to be higher for P450 2A6 than P450 2A13 (10 fold) by He et al. [85], while Weymarn and Murphy found that both enzymes had similar efficiency [86]. DeVore et al. measured spectral binding affinities (*K_D_*) for coumarin and found equal binding affinities to both P450s 2A6 and 2A13 [87]. The planar active site and Asn297 are conserved in both enzymes. DeVore et al. performed receptor-based QSAR studies using the comparative binding energy (COMBINE) approach and identified individual protein/ligand interactions. These studies revealed that the binding orientation was similar for both proteins with coumarin forming a hydrogen bond to Asn297. This was consistent with both enzymes generating 7-hydroxycoumarin with identical *K_D_* values. While P450 2A6 exclusively formed 7-hydroxycoumarin, von Weymarn and Murphy [86] found that P450 2A13 formed significant amounts of 3,4-epoxide and smaller amounts of 6-hydroxycoumarin and 8-hydroxycoumarin in addition to 7-hydroxycoumarin. DeVore et al. found that the approach path of coumarin substrate to the activated oxygen on the heme was less constrained in P450 2A13 than in P450 2A6, leading to hydroxylation at the 6- or 8- positions, and also the inverted orientation of coumarin that resulted in 3,4-eopxide [87]. Comparison of the active site residues between P450s 2A6 and 2A13 revealed that 10 out of 32 residues differ between the two enzymes, potentially correlating to the functional differences in substrate metabolism. The COMBINE QSAR model generated by DeVore et al. suggested that steric roles played by the active site residues were primarily responsible for the differing ligand affinities [87].

Homology models of rat and human P450 2D isozymes (2D1, 2D2, 2D3, 2D4, and 2D6) have been built and studied [61]. Docking followed by molecular dynamic simulations were carried on 7-methoxy-4-aminomethylcoumarin [61]. The models showed that the Asp301 residue of these enzymes form hydrogen bonds with the basic nitrogen of 7-methoxy-4-aminomethylcoumarin, resulting in its O-demethylation [61].

**Figure 11:**
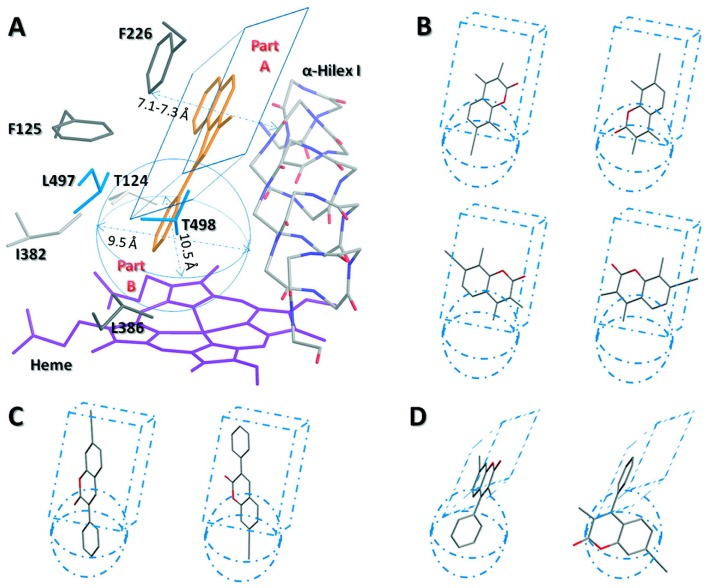
Reprinted with permission from reference 22. (**A**) Diagram of the active site cavity of P450 1A2: a narrow, but extended channel (part **A**), and a spherical hydrophobic pocket (part **B**). (**B**) The binding patterns of compound 7ETMC with P450 1A2, which suggests that the midsize 7-ethynylcoumarins could freely rotate in the narrow cavity (part **A**) if only it is parallel with Phe-226 and the peptide bond in the α-helix I in order to form π–π interactions. (**C**) The possible binding patterns of compound 7E3PC with P450 1A2. Phenyl-facing-heme orientation (left) takes an obvious priority over the acetylene-facing-heme orientation (right). (**D**) The possible binding pattern (left) and impossible binding pattern (right) of compound 7E3M4PC with P450 1A2, suggesting that there is no reactive pose for the docking of 7E3M4PC with this enzyme.

Docking studies were performed in our laboratory for a series of ethynylcoumarins with P450 enzymes 1A1 and 1A2 using the LigandFit in the Accelrys DS studio^®^ (Figure 11) [22]. These studies showed that π-π interactions of the coumarins with the phenylalanine residues of the active site are critical determinants of the binding mode of the inhibitors. Changes in orientations guided by the position of the phenyl substituents dictate the nature of inhibition as a competitive or time-dependent inhibitor of the P450 1A2 enzyme. In the absence of a phenyl substituent, the ethynyl groups were the closest to the heme-Fe, enabling the generation of a highly reactive oxidation intermediate that could then react with the receptor residues to form covalent interactions leading to a time-dependent inhibition and inactivation of the enzyme. The presence of a 3-phenyl substituent on the coumarin ring flipped the molecule’s orientation in the active site to facilitate the phenyl group’s facing the heme-Fe, and the ethynyl group facing away from the heme. This leads to these molecules acting as competitive inhibitors of P450 1A2. P450 1A1 has a larger active site cavity in comparison to P450 1A2. This results in multiple binding modes for the ethynyl coumarin series studied leading to some of the binding modes orienting the ethynyl group in close proximity to the heme-Fe causing time-dependent inhibition by all of these compounds. QSAR studies were performed on a series of 7-coumarin propargyl ethers using comparative molecular field analysis (CoMFA), comparative molecular similarity index analysis (CoMSIA) and hologram quantitative structure-activity relationship (HQSAR) modules in Tripos-Sybyl [19]. These analyses showed that there are equal contributions from steric, electrostatic and ClogP descriptors towards the inhibition potency of these compounds toward P450 1A2, indicating that lipophilicity is an important factor. The contour maps of steric and electrostatic plots were generated for the binding pocket of P450 1A2 [19]. Lewis et al. performed QSAR analysis on a series of 7-alkoxycoumarins that are used as diagnostic probes for the P450 2B enzymes [84]. The logP data which is a lipophilicity index was obtained experimentally and by calculations via the ClogP. They found a quadratic relationship between the logP of 7-alkoxycoumarins and the experimentally determined logarithm of the dealkylation activity in phenobarbital-induced animals. Hydrophobicity was found to be a key factor in the P450 2B and substrate interactions. Additional interactions such a hydrogen bonding and pi-pi interactions were also found to be relevant.

Leong et al. used a hierarchical support vector regression (HVSR) approach to predict the interactions of several drugs with P450 2B6 [88]. Cytochrome P450 2B6 is a hepatic enzyme constituting 1% of the total hepatic P450 complement [89], but metabolizes 3% of the clinical drugs [90]. Five coumarins, 7-ethoxycoumarin, 7-ethoxy-4-trifluoromethylcoumarin, 7-methoxy-4-trifluoromethylcoumarin, 3-cyano-7-ethoxycoumarin, and 4-chloromethyl-7-ethoxycoumarin were used in this study [88]. The generated HVSR model showed a relation between the experimental and predicted values, which were statistically true (correlation coefficient *q*^2^ of 0.93 and *r*^2^ values of 0.97 and 0.82 for the training set and test set, respectively for the 10-cross validation of the model) [88]. Out of a total of 10 descriptors, logP’s were shown to be of critical importance, and the descriptors relating to the molecular size and shape were also important contributors to the model [88].

The docking and QSAR studies by various research groups have provided a deeper understanding of the nature and types of interactions of coumarins with the active site residues of P450 1A, 1B, 2B, 2C, 2D and 3A enzymes.

## 2. Conclusions

Structure-activity relationship studies through bioassays and computational molecular modeling have provided important insights into the interaction patterns and mechanisms of the action of cytochrome P450 enzymes with their substrates and inhibitors. These studies have led to important findings about the structural features required for substrate metabolism and/or inhibition of these enzymes. Docking studies and molecular dynamic simulations of protein-ligand docked complexes have been performed. Docking studies have shown the importance of π-π interactions between coumarins and the phenylalanine residues in the active site, along with important hydrogen-bonding residues that can change the binding mode of the coumarins. The optimum distances between the heme-iron and the site of oxidation on the substrates for the various P450 enzymes have also been determined through docking studies. Ligand-based studies such as QSAR studies and structural meta-analysis studies have shown that three descriptors- steric, electrostatic, and ClogP play critical roles in determining the inhibition potency of the coumarins toward P450 1A2.

## References

[1] Guengerich F.P., Ortiz de Montellano P.R. (2005). Cytochrome P450 Structure, Mechanism, and Biochemistry.

[2] Hasemann C.A., Kurumbail R.G., Boddupalli S.S., Peterson J.A., Deisenhofer J. (1995). Structure and function of cytochromes P450: A comparative analysis of three crystal structures. Curr. Biol..

[3] Capdevila J.H., Zeldin D., Makita K., Karara A., Falck J.R., Ortiz de Montellano P.R. (2005). Cytochrome P450 Structure, Mechanism, and Biochemistry.

[4] Williams P.A., Cosme J., Sridhar V., Johnson E.F., McRee D.E. (2000). Mammalian microsomal cytochrome P450 monooxygenase: Structural adaptations for membrane binding and functional diversity. Mol. Cell.

[5] Schenkman J.B., Greim H. (1993). Cytochrome P450 Handbook of Experimental Pharmacology.

[6] Williams J.A., Hyland R., Jones B.C., Smith D.A., Hurst S., Goosen T.C., Peterkin V., Koup J.R., Ball S.E. (2004). Drug-drug interactions for UDP-glucuronosyltransferase substrates: A pharmacokinetic explanation for typically observed low exposure (AUC /AUC) ratios. Drug Metab. Dispos..

[7] Guengerich F.P., Kim D.-H., Iwasaki M. (1991). Enzymatic oxidation of ethyl carbamate to vinyl carbamate and its role as an intermediate in the formation of 1, N6-ethenoadenosine. Chem. Res. Toxicol..

[8] Zhang J.Y., Wang Y., Prakash C. (2006). Xenobiotic-metabolizing enzymes in human lung. Curr. Drug Metab..

[9] Shimada T., Hayes C.L., Yamazaki H., Amin S., Hecht S.S., Guengerich F.P., Sutter T.R. (1996). Activation of chemically diverse procarcinogens by human cytochrome P450 1B1. Cancer Res..

[10] Shimada T., Gillam E.M., Sandhu P., Guo Z., Tukey R.H., Guengerich F.P. (1994). Activation of procarcinogens by human cytochrome P450 enzymes expressed in *Escherichia coli*. Simplified bacterial systems for genotoxicity assays. Carcinogenesis.

[11] Shimada T., Oda Y., Gillam E.M.J., Guengerich F.P., Inoue K. (2001). Metabolic activation of polycyclic aromatic hydrocarbons and other procarcinogens by cytochromes P450 1A1 and P450 1B1 allelic variants and other human cytochromes P450 in Salmonella typhimurium NM2009. Drug Metab. Dispos..

[12] Nelson D.R. (2011). Progress in tracing the evolutionary paths of cytochrome P450. Biochim. Biophys. Acta BBA Proteins Proteom..

[13] Liu J., Taylor S.F., Dupart P.S., Arnold C.L., Sridhar J., Jiang Q., Wang Y., Skripnikova E.V., Zhao M., Foroozesh M. (2013). Pyranoflavones: A group of small-molecule probes for exploring the active site cavities of cytochrome P450 enzymes 1A1, 1A2, and 1B1. J. Med. Chem..

[14] Liu J., Sridhar J., Foroozesh M. (2013). Cytochrome P450 family 1 inhibitors and structure-activity relationships. Molecules.

[15] Sridhar J., Liu J., Foroozesh M., Klein Stevens C.L. (2012). Inhibition of cytochrome P450 enzymes by quinones and anthraquinones. Chem. Res. Toxicol..

[16] Sridhar J., Liu J., Komati R., Schroeder R., Jiang Q., Tram P., Riley K., Foroozesh M. (2016). Ortho-methylarylamines as time-dependent inhibitors of cytochrome P450 1A1 enzyme. Drug Metab. Lett..

[17] Sridhar J., Liu J., Foroozesh M., Stevens C.L. (2012). Insights on cytochrome P450 enzymes and inhibitors obtained through QSAR studies. Molecules.

[18] Sridhar J., Ellis J., Dupart P., Liu J., Stevens C.L., Foroozesh M. (2012). Development of flavone propargyl ethers as potent and selective inhibitors of cytochrome P450 enzymes 1A1 and 1A2. Drug Metab. Lett..

[19] Sridhar J., Foroozesh M., Stevens C.L. (2011). QSAR models of cytochrome P450 enzyme 1A2 inhibitors using CoMFA, CoMSIA and HQSAR. SAR QSAR Environ. Res..

[20] Sridhar J., Jin P., Liu J., Foroozesh M., Stevens C.L. (2010). In silico studies of polyaromatic hydrocarbon inhibitors of cytochrome P450 enzymes 1A1, 1A2, 2A6, and 2B1. Chem. Res. Toxicol..

[21] Shimada T., Tanaka K., Takenaka S., Murayama N., Martin M.V., Foroozesh M.K., Yamazaki H., Guengerich F.P., Komori M. (2010). Structure-function relationships of inhibition of human cytochromes P450 1A1, 1A2, 1B1, 2C9, and 3A4 by 33 flavonoid derivatives. Chem. Res. Toxicol..

[22] Liu J., Nguyen T.T., Dupart P.S., Sridhar J., Zhang X., Zhu N., Stevens C.L., Foroozesh M. (2012). 7-ethynylcoumarins: Selective inhibitors of human cytochrome P450s 1A1 and 1A2. Chem. Res. Toxicol..

[23] Sridhar J., Goyal N., Liu J., Foroozesh M. (2017). Review of ligand specificity factors for CYP1A subfamily enzymes from molecular modeling studies reported to-date. Molecules.

[24] Walsh A.A., Szklarz G.D., Scott E.E. (2013). Human cytochrome P450 1A1 structure and utility in understanding drug and xenobiotic metabolism. J. Biol. Chem..

[25] Androutsopoulos V.P., Tsatsakis A.M., Spandidos D.A. (2009). Cytochrome P450 CYP1A1: Wider roles in cancer progression and prevention. BMC Cancer.

[26] Williams P.A., Cosme J., Vinkovic D.M., Ward A., Angove H.C., Day P.J., Vonrhein C., Tickle I.J., Jhoti H. (2004). Crystal structures of human cytochrome P450 3A4 bound to metyrapone and progesterone. Science.

[27] Wang A., Savas U., Hsu M.-H., Stout C.D., Johnson E.F. (2012). Crystal structure of human cytochrome P450 2D6 with prinomastat bound. J. Biol. Chem..

[28] Bart A.G., Scott E.E. (2018). Structures of human cytochrome P450 1A1 with bergamottin and erlotinib reveal active-site modifications for binding of diverse ligands. J. Biol. Chem..

[29] Yun C.-H., Miller G.P., Guengerich F.P. (2001). Oxidations of p-alkoxyacylanilides catalyzed by human cytochrome P450 1A2: Structure-activity relationships and simulation of rate constants of individual steps in catalysis. Biochemistry.

[30] Yano J.K., Hsu M.-H., Griffin K.J., Stout C.D., Johnson E.F. (2005). Structures of human microsomal cytochrome P450 2A6 complexed with coumarin and methoxsalen. Nat. Struct. Mol. Biol..

[31] Shah M.B., Liu J., Huo L., Zhang Q., Dearing M.D., Wilderman P.R., Szklarz G.D., Stout C.D., Halpert J.R. (2016). Structure-function analysis of mammalian CYP2B enzymes using 7-substituted coumarin derivatives as probes: Utility of crystal structures and molecular modeling in understanding xenobiotic metabolism. Mol. Pharmacol..

[32] Pereira T.M., Franco D.P., Votorio F., Kummerle A.E. (2018). Coumarin compounds in medicinal chemistry: Some important examples from the last years. Curr. Top. Med. Chem..

[33] Poumale H.M., Hamm R., Zang Y., Shiono Y., Kuete V., Kuete V. (2013). Medicinal Plant Research in Africa, Pharmacology and Chemistry.

[34] Bone K., Mills S. (2013). Principles and Practice of Phytotherapy.

[35] Aoyama Y., Katayama T., Yamamoto M., Tanaka H., Kon K. (1992). A new antitumor antibiotic product, demethylchartreusin. Isolation and biological activities. J. Antibiot..

[36] Venugopala K.N., Rashmi V., Odhav B. (2013). Review on natural coumarin lead compounds for their pharmacological activity. BioMed Res. Int..

[37] Born S.L., Caudill D., Filter K.L., Purdonm M.P. (2002). Identification of the cytochromes P450 that catalyze coumarin 3, 4-epoxidation and 3-hydroxylation. Drug Metab. Dispos..

[38] Sridar C., Kent U.M., Noon K., McCall A., Alworth W., Foroozesh M., Hollenberg P.F. (2008). Differential inhibition of cytochromes P450 3A4 and 3A5 by the newly synthesized coumarin derivatives 7-coumarin propargyl ether and 7-(4-trifluoromethyl) coumarin propargyl ether. Drug Metab. Dispos..

[39] Lewis D.F., Ito Y., Lake B.G. (2006). Metabolism of coumarin by human P450s: A molecular modelling study. Toxicol. In Vitro.

[40] Lake B.G. (1999). Coumarin metabolism, toxicity and carcinogenicity: Relevance for human risk assessment. Food Chem. Toxicol..

[41] Bourgaud F., Hehn A., Larbat R., Doerper S., Gontier E., Kellner S., Matern U. (2006). Biosynthesis of coumarins in plants: A major pathway still to be unraveled for cytochrome P450 enzymes. Phytochem. Rev..

[42] He K., Lyer K.R., Hayes R.N., Sinz M.W., Woolf T.F., Hollenberg P.F. (1998). Inactivation of cytochrome P450 3A4 by bergamottin, a component of grapefruit juice. Chem. Res. Toxicol..

[43] Lin H.L., Kent U.M., Hollenberg P.F. (2005). The grapefruit juice effect is not limited to cytochrome P450 (P450) 3A4: Evidence for bergamottin-dependent inactivation, heme destruction, and covalent binding to protein in P450s 2B6 and 3A5. J. Pharmacol. Exp. Ther..

[44] Hung W.-L., Suh J.H., Wang Y. (2017). Chemistry and health effects of furanocoumarins in grapefruit. J. Food Drug Anal..

[45] Kleiner H.E., Xia X., Sonoda J., Zhang J., Pontius E., Abey J., Evans R.M., Moore D.D., DiGiovanni J. (2008). Effects of naturally occurring coumarins on hepatic drug-metabolizing enzymes in mice. Toxicol. Appl. Pharmacol..

[46] Tine M., Belghit J., Descatoire V., Amouyal G., Letteron P., Geneve J., Larrey D., Pessayre D. (1987). Inactivation of human liver cytochrome P-450 by the drug methoxsalen and other psoralen derivatives. Biochem. Pharmacol..

[47] Palacharla R.C., Molgara P., Panthangi H.R., Boggavarapu R.K., Manoharan A.K., Ponnamaneni R.K., Ajjala D.R., Nirogi R. (2017). Methoxsalen as an in vitro phenotyping tool in comparison with 1-aminobenzotriazole. Xenobiotica.

[48] Prince M., Campbell C.T., Robertson T.A., Wells A.J., Kleiner H.E. (2006). Naturally occurring coumarins inhibit 7, 12-dimethylbenz [a] anthracene DNA adduct formation in mouse mammary gland. Carcinogenesis.

[49] Born S.L., Hu J.K., Lehman-McKeeman L.D. (1999). O-Hydroxyphenylacetaldehyde is a hepatotoxic metabolite of coumarin. Drug Metab. Dispos..

[50] Zanger U.M., Schwab M. (2013). Cytochrome P450 enzymes in drug metabolism: Regulation of gene expression, enzyme activities, and impact of genetic variation. Pharmacol. Ther..

[51] Crespi C.L., W-Penman B., Steimel D.T., Smith T., Yang C.S., Sutter T.R. (1997). Development of a human lymphoblastoid cell line constitutively expressing human CYP1B1 cDNA: Substrate specificity with model substrates and promutagens. Mutagenesis.

[52] Yun C.-H., Kim K.-H., Calcutt M.W., Guengerich F.P. (2005). Kinetic analysis of oxidation of coumarins by human cytochrome P450 2A6. J. Biol. Chem..

[53] Miwa G.T., Lu A.Y.H. (1987). Kinetic isotope effects and “metabolic switching” in cytochrome P450-catalyzed reactions. BioEssays.

[54] Miwa G.T., Walsh J.S., Lu A.Y.H. (1984). Kinetic isotope effects on cytochrome P-450-catalyzed oxidation reactions: The oxidative *O*-dealkylation of 7-ethoxycoumarin. J. Biol. Chem..

[55] Liu J., Shah M.B., Zhang Q., Stout C.D., Halpert J.R., Wilderman P.R. (2016). Coumarin derivatives as substrate probes of mammalian cytochromes P450 2B4 and 2B6: Assessing the importance of 7-alkoxy chain length, halogen substitution, and non-active site mutations. Biochemistry.

[56] Scott E.E., White M.A., He Y.A., Johnson E.F., Stout C.D., Halpert J.R. (2004). Structure of mammalian cytochrome P450 2B4 complexed with 4-(4-Chlorophenyl)imidazole at 1.9-Å resolution. J. Biol. Chem..

[57] Shah M.B., Pascual J., Zhang Q., Stout C.D., Halpert J.R. (2011). Structures of cytochrome P450 2B6 bound to 4-benzylpyridine and 4-(4-nitrobenzyl)pyridine: Insight into inhibitor binding and rearrangement of active site side chains. Mol. Pharmacol..

[58] Nahar R., Dube D.P., Parakh R., Deb R., Saxena R.S., Singh T.P., Verma I.C. (2013). Implication of novel CYP2C9*57 (p. Asn204His) variant in coumarin hypersensitivity. Thromb. Res..

[59] Ngui J.S., Chen Q., Shou M., Wang R.W., Stearns R.A., Baillie T.A., Tang W. (2001). In vitro stimulation of warfarin metabolism by quinidine: Increases in the formation of 4′- and 10-hydroxywarfarin. Drug Metab. Dispos..

[60] Zanger U.M., Fischer J., Raimundo S., Stüven T., Evert B.O., Schwab M. (2001). Comprehensive analysis of the genetic factors determining expression and function of hepatic CYP2D6. Pharmacogenetics.

[61] Venhorst J., Laak A.M., Commandeur J.N., Hiroi T., Vermeulen N.P. (2003). Homology modeling of rat and human cytochrome P450 2D (CYP2D) isoforms and computational rationalization of experimental ligand-binding specificities. J. Med. Chem..

[62] Yamazaki H., Inoue K., Mimura M., Oda Y., Guengerich F.P., Shimada T. (1996). 7-Ethoxycoumarin O-deethylation catalyzed by cytochromes P450 1A2 and 2E1 in human liver microsomes. BioChem. Pharmacol..

[63] Wei Y., Wu H., Li L., Liu Z., Zhou X., Zhang Q.-Y., Weng Y., D’Agostino J., Ling G., Zhang X. (2012). Generation and Characterization of a *CYP2A13*/*2B6*/*2F1*- Transgenic Mouse Model. Drug Metab. Dispos..

[64] Mooiman K.D., Maas-Bakkar R.F., Hendrikx J.J.M.A., Bank P.C.D., Rosing H., Beijnen J.H., Schellens J.H.M., Meijerman I. (2014). The effect of complementary and alternative medicines on CYP3A4—Mediated metabolism of three different substrates: 7-Benzyloxy-4-trifluoromethyl--coumarin, midazolam and docetaxel. J. Pharm. Pharmacol..

[65] Rwei A.Y., Wang W., Kohane D.S. (2015). Photoresponsive nanoparticles for drug delivery. Nano Today.

[66] Wagner N., Stephan M., Hoglinger D., Nadler A. (2018). A click case: Organelle-specific uncaging of lipid messengers. Angew. Chem. Int. Ed..

[67] Yang L., Tang H., Sun H. (2018). Progress in photo-responsive polypeptide derived nano-assemblies. Micromachines.

[68] Schmidt R., Geissler D., Hagen V., Bendig J. (2007). Mechanism of photocleavage of (coumarin-4-yl) methyl esters. J. Phys. Chem. A.

[69] Lin Q., Bao C., Cheng S., Yang Y., Ji W., Zhu L. (2012). Target-activated coumarin phototriggers specifically switch on fluorescence and photocleavage upon binding to thiol-bearing protein. J. Am. Chem. Soc..

[70] Lin Q., Yang L., Wang Z., Hua Y., Zhang D., Bao B., Bao C., Gong X., Zhu L. (2019). Coumarin photocages modified with an electron-rich styryl moiety at the 3-position: Long wavelength excitation, rapid photolysis and photobleaching. Angew. Chem. Int. Ed..

[71] Ueng Y.F., Jan W.C., Lin L.C., Chen T.L., Guengerich F.P., Chen C.F. (2002). The alkaloid rutaecarpine is a selective inhibitor of cytochrome p450 1a in mouse and human liver microsomes. Drug Metab. Dispos..

[72] Davies H.W., Britt S.G., Pohl L.R. (1986). Inactivation of cytochrome p-450 by 2-isopropyl-4-pentenamide and other xenobiotics leads to heme-derived protein adducts. Chem. Biol. Interact..

[73] Halpert J. (1982). Further studies of the suicide inactivation of purified rat liver cytochrome p-450 by chloramphenicol. Mol. Pharmacol..

[74] Liu J., Pham P.T., Skripnikova E.V., Zheng S., Lovings L.J., Wang Y., Goyal N., Bellow S.M., Mensah L.M., Chatters A.J. (2015). A ligand-based drug design. Discovery of 4-trifluoromethyl-7, 8-pyranocoumarin as a selective inhibitor of human cytochrome P450 1A2. J. Med. Chem..

[75] Rendic S., Guengerich F.P. (2012). Contributions of human enzymes in carcinogen metabolism. Chem. Res. Tocixol..

[76] Shimada T. (2017). Inhibition of Carcinogen-activating cytochrome P450 enzymes by xenobiotic chemicals in relation to antimutagenicity and anticarcinogenecity. Toxicol. Res..

[77] Yamaguchi T., Akimoto I., Motegi K., Yoshimura T., Wada K., Nishizono N., Oda K. (2013). Synthetic models related to methoxalen and menthofuran-cytochrome P450 (CYP) 2A6 interactions. benzofuran and coumarin derivatives as potent and selective inhibitors of CYP2A6. Chem. Pharm. Bull..

[78] Meineke I., Desel H., Kahl R., Kahl G.F., Gundert-Remy U. (1998). Determination of 2-hydroxyphenylacetic acid (2HPAA) in urine after oral and parenteral administration of coumarin by gas-liquid chromatography with flame-ionization detection. J. Pharm. Biomed. Anal..

[79] Pelkonen O., Rautio A., Raunio H., Pasanen M. (2000). CYP2A6: A human coumarin 7-hydroxylase. Toxicology.

[80] Rautio A., Kraul H., Koji A., Salmela E., Pelkonen O. (1992). Interindividual variability of coumarin 7-hydroxylase in healthy volunteers. Pharmacogenetics.

[81] Van Iersel M.L.P.S., Henderson C.J., Walters D.G., Price R.J., Wolf C.R., Lake B.G. (1994). Metabolism of [3-^14^C] coumarin by human liver microsomes. Xenobiotica.

[82] Zhuo X., Gu J., Zhang Q.-Y., Spink D.C., Kaminsky L.S., Ding X. (1999). Biotransformation of coumarin by rodent and human cytochromes P450: Metabolic basis of tissue-selective toxicity in olfactory mucosa of rats and mice. J. Pharmacol. Exp. Ther..

[83] Lewis D.F.V., Lake B.G., Dickins M. (2007). Quantitative structure-activity relationships (QSARs) in inhibitors of various cytochromes P450: The importance of compound lipophilicity. J. Enzym. Inhib. Med. Chem..

[84] Lewis D.F.V., Ito Y., Lake B.G. (2010). Quantitative structure-activity relationships (QSARs) for inhibitors and substrates of CYP2B enzymes: Importance of compound lipophilicity in explanation of potency differences. J. Enzym. Inhib. Med. Chem..

[85] He X.Y., Shen J., Hu W.Y., Ding X., Lu A.Y., Hong J.Y. (2004). Identification of Val117 and Arg372 as critical amino acid residues for the activity difference between human CYP2A6 and CYP2A13 in coumarin 7-hydroxylation. Arch. Biochem. Biophys..

[86] Von Weymarn L.B., Murphy S.E. (2003). CYP2A13-catalysed coumarin metabolism: Comparison with CYP2A5 and CYP2A6. Xenobiotica.

[87] DeVore N.M., Smith B.D., Wang J.L., Lushington G.H., Scott E.E. (2009). Key residues controlling binding of diverse ligands to human cytochrome P450 2A enzymes. Drug Metab. Dispos..

[88] Leong M.K., Chen Y.M., Chen T.-H. (2009). Prediction of human cytochrome P450 2B6-substrate interactions using Hierarchical Support Vector Regression approach. J. Comput. Chem..

[89] Shimada T., Yamazaki H., Mimura M., Inui Y., Guengerich F.P. (1994). Interindividual variations in human liver cytochrome P-450 enzymes involved in the oxidation of drugs, carcinogens and toxic chemicals: Studies with liver microsomes of 30 Japanese and 30 Caucasians. J. Pharmacol. Exp. Ther..

[90] Rendic S., DiCarlo F.J. (1997). Human cytochrome P450 enzymes: A status report summarizing their reactions, substrates, inducers, and inhibitors. Drug Metab. Rev..

